# Selenium deficiency negatively affects survival and integrity of human hippocampal progenitor cells^☆^

**DOI:** 10.1016/j.nbas.2025.100138

**Published:** 2025-04-30

**Authors:** Sahand Farmand, Emaan Ahmed, Hadisa Azizi Zawar, Sandrine Thuret

**Affiliations:** Department of Basic and Clinical Neuroscience, Institute of Psychiatry, Psychology and Neuroscience, King’s College London, London, United Kingdom

**Keywords:** Adult hippocampal neurogenesis, Selenium, Neural stem cells, Aging, Cognition

## Abstract

Selenium has been shown to be a key regulatory element in the health, survival and proliferation of neural stem and progenitor cells, with various studies underlining its anti-aging properties. However, most of this knowledge is derived from rodent models, leaving its effects on human hippocampal progenitor cells unclear. In this study, we utilized a human hippocampal progenitor cell (HPC) line to examine the effects of varying concentrations of sodium selenite, an inorganic form of selenium (0 µM, 0.1 µM, 0.23 µM, 0.5 µM, and 1.0 µM), on the proliferation, apoptosis, and progenitor integrity of these cells. To do this, HPCs were exposed to these concentrations for 48 h, followed by immunocytochemistry to quantify, cell number (DAPI-positive cells), proliferation (KI67-positve cells), apoptosis (CC3-positve cells), and progenitor integrity (SOX2- and Nestin-positive cells). While our results indicated no significant effects of selenium concentrations on proliferation or apoptosis, we demonstrated that absence of selenium (0 μM) in the culture media significantly reduced both cell number and percentage of Nestin-positive cells, but only when compared to the condition with the highest selenium concentration (1.0 μM). Our findings underscore the role of selenium in regulating the survival and integrity of human HPCs. Lastly, we emphasize the need for further research to uncover the mechanisms underlying these observed changes.

## Introduction

Adult hippocampal neurogenesis (AHN)—a neuroplastic process during which neural stem cells (NSCs), located within the subgranular zone (SGZ) of the dentate gyrus (DG), give rise to neurons [1,2]—has been shown to play a vital role in certain aspects of hippocampal-dependent cognition, including pattern separation [3,4] and learning and memory [5]. Similar to cognition, AHN has also been shown to be negatively affected by aging. For instance, rodent studies [6] as well as post-mortem analysis of human brains [7,8] have revealed an age-dependent reduction in numbers of NSCs as well as neural progenitor cells (NPCs). While the exact mechanisms of the age-related impairment of AHN remain under investigation, aging is speculated to induce reduced proliferative capacity [9,10], deep quiescence [11,12] and poor survival of NSCs [6,13].

Various rodent and human studies have demonstrated that physical exercise (PE), can help maintain hippocampal volume [14,15], regulate AHN [16] and enhance hippocampal-dependent cognition [14,17], underscoring its potential as a strategy to combat age-related cognitive decline (For detailed review, see [18]). However, the underlying mechanisms through which PE influences AHN—and, consequently, hippocampal-dependent cognition—are not yet well understood. Different human and rodent studies have indicated that exercise-induced expression and secretion of growth factors, such as brain-derived neurotrophic factor [19,20], vascular endothelial growth factor [21,22], and insulin like growth factor-1 [23], both locally within the hippocampus and systemically through the bloodstream, play a critical role in this process.

In support of the role of blood-borne factors in regulating AHN, [24] demonstrated that NSCs within the neurogenic niche are directly in contact with blood-borne factors via vesicular transcytosis, suggesting that any potential upregulations or downregulations of blood content can affect NSCs. Aiming to identify the blood factors that mediate PE’s beneficial effects, Leiter et al. [25] showed that post-PE plasma collected from mice, had significantly higher levels of Selenoprotein P (SEPP1), a key protein involved in selenium retention and transportation via its receptor low density lipoprotein-receptor like 8 (LRP8), also referred to as apolipoprotein E receptor 2 (apoER2). In fact, selenium has been shown to be a vital trace element essential for healthy aging due to its critical role in regulating oxidative stress and supporting cellular defence mechanisms [26]. Furthermore, speculating that selenium potentially contributes to enhanced AHN, Leiter et al. [27] demonstrated that ex vivo supplementation with sodium selenite (SS), an inorganic form of selenium, promotes neurospheres formation in primary DG cells and significantly boosts the proliferation of adherent hippocampal-precursor cells. In the same study, direct infusion of SS into the mouse hippocampus led to a significant increase in the proportion of proliferating cells located within the SGZ, while boosting the survival of proliferating cells by 50 %. Importantly, Leiter et al., [27] also demonstrated that knockout of the *SEPP1* and *LRP8* genes abolishes the beneficial impact of PE on AHN, highlighting the potential role of selenium and its receptor, LRP8, in PE-induced AHN.

While the role of selenium in mediating the beneficial effects of PE on AHN and cognition has been established in mice, it remains unknown whether selenium levels increase following exercise in humans and whether increased levels of selenium can promote the proliferation of human NSCs/NPCs. To explore this, here, by using an in vitro model of hippocampal neurogenesis, we set out to investigate how selenium supplementation effects human hippocampal progenitor cells (HPCs), with a specific focus on cell proliferation, apoptosis and progenitor cell integrity.

## Materials and methods

The authors declare that ethical standards prevailing at their institutions have been observed throughout this study.

### Cell culture and maintenance

Human hippocampal progenitor cell line (HPC0A07/03A; ReNeuron LTD., Surrey, United Kingdom), derived from the brain of a first-trimester female foetus −in accordance with UK and USA ethical and legal guidelines- were used throughout this study. HPCs were immortalised with c*-mycER^TAM^* gene to conditionally proliferate in presence of 100 nM 4-hydroxy-tamoxifen (4-OHT, Sigma Aldrich, #H7904), 10 ng/mL human basic fibroblast growth factor (bFGF, Peprotech, #100-18B-500) and 20 ng/mL human epidermal growth factor (EGF, Peprotech, #AF100-15–500) and spontaneously differentiate (Supplementary Fig. S1) in their absence, as described before [[28], [29], [30], [31]]. Cells were cultured in filtered Dulbecco’s Modified Eagle’s Media/F12 (DMEM:F12, Gibco, #21331046), consisting of 0.03 % human albumin solution (Octapharma, #5111903), 100 μg/mL human apo-transferrin (Sigma Aldrich, #T1147), 15 mM HEPES (Gibco, #15630056), 16.2 μg/mL human putrescine diHCl (Sigma Aldrich, #P5780), 5 μg/mL human recombinant insulin (Sigma Aldrich, #I9278), 60 ng/mL progesterone (Sigma Aldrich, #P8783), 2 mM L-glutamine (Sigma Aldrich, #G7513) and 40 ng/mL sodium selenite (Sigma Aldrich, #S9133-1MG). HPCs were maintained on laminin (Gibco, #23017015) coated culture vessels (Nunclon, Denmark) and passaged at 80–90 % confluency. To do this, HPCs were initially washed with PBS (Gibco, #18912014) and detached using Accutase (Gibco, #00–4555-56). Cell counting was then performed using trypan blue (Sigma Aldrich, #T8154) to distinguish between live and dead cells. Finally, cells were seeded and incubated at 37 °C, 5 % CO2, before being plated for experiments. Cells used in this project were between passage 21 to passage 23.

### In vitro supplementation of proliferating human HPCs with sodium selenite

**Fig. 1:**
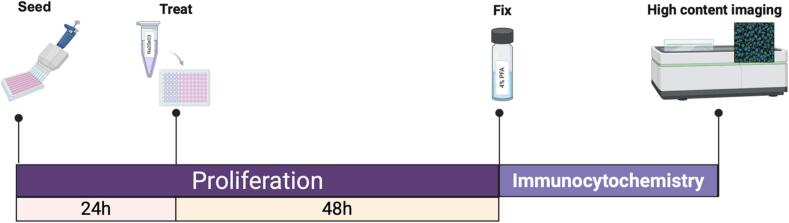
Schematic of the proliferation assays for in vitro supplementation of *HPC0A07/03A* cells with sodium selenite. To assess markers associated with proliferation, apoptosis and progenitor cell integrity using immunocytochemistry, cells were seeded in sodium selenite-free media and allowed to proliferate for 24 h. The sodium selenite-free media was then removed from the wells, and cells were treated with various concentrations of sodium selenite (0 μM, 0.1 μM, 0.23 μM, 0.5 μM and 1.0 μM). After a further 48 h of proliferation, cells were fixed using 4 % PFA, immunocytochemistry was performed, followed by high content imaging using the Opera Phenix. (Created with BioRender).

The proliferation assay was conducted as previously described [28,32]. Since the media used for culturing HPCs normally contains SS, HPCs were initially seeded in SS-free media in 96-well plates (Nunclon, Denmark) at a seeding density of 1.3 × 10^4^ cells per well. 24 h post seeding, the SS-free media was removed, and cells were treated with media containing varying concentrations of SS (0 μM, 0.1 μM, 0.23 μM, 0.5 μM and 1.0 μM; range selected based on [27]). The cells were then allowed to proliferate for an additional 48 h, bringing the total proliferation time to 72 h (See timeline/workflow; Fig. 1). Each condition included three biological replicates (*n = 3*), with three technical replicates per biological replicate (*n = 3*).

### Immunocytochemistry

As previously described [28,32], 72 h after seeding (48 h post-treatment), HPCs were fixed in 4 % paraformaldehyde (PFA) for 20 min at room temperature. Cells were then blocked for 1 h in a blocking solution containing phosphate-buffered saline (PBS), 5 % normal donkey serum (Sigma Aldrich, #D9963), and 0.3 % Triton-X-100 (Sigma Aldrich, #93443). Following blocking, cells were incubated overnight at 4 °C with primary antibodies (see Table 1. for antibody details). The next day, cells were incubated with secondary antibodies for 2 h at room temperature (see; Table 1.). Finally, nuclei were stained by incubating cells with 300 µM DAPI (Sigma Aldrich, #D9542-5 mg) for 5 min at room temperature.

**Table 1:** List of antibodies used in the study, species, source, catalogue number and dilution factor. Ki67: antigen Kiel-67, CC3: Cleaved Caspase-3, Nestin: neuroepithelial stem cell protein and SOX2: Sex-determining Region Y-Box 2.

Antibody	Source	Catalogue number	Dilution	Marker for
Mouse Anti-ki67	Cell Signalling	9449	1:800	Proliferation
Rabbit Anti-CC3	Invitrogen	PA5-114687	1:1000	Apoptosis
Mouse Anti-Nestin	Merck	MAB5326	1:1000	Stem cell integrity
Rabbit Anti-SOX2	Merck	AB5603	1:1000	Stem cell integrity
555 Donkey Anti-Rabbit	Life-Technologies	A-31572	1:500	–
488 Donkey Anti-Mouse	Life-Technologies	A-21202	1:500	–

### High-content imaging and image analysis

Imaging was performed using the Opera Phenix high-content screening system (PerkinElmer, USA) with a 20× water objective. DAPI fluorescence was detected using a 385 nm laser, while secondary antibody fluorescence was captured with 488 nm and 568 nm lasers. Image analysis was conducted with Harmony software (version 4.9; PerkinElmer). Once these threshold settings as well as parameters based on the cell size and shape were optimized for imaging and analysis, these settings were held constant across all experiments to ensure consistency.

### Statistical analysis

Data analysis was performed using RStudio (Version 2024.04.2 + 764). Prior to analysis, all data were evaluated for normality and homogeneity of variance using the Shapiro-Wilk test and Levene's test, respectively. Data that were normally distributed and met the assumption of homogeneity of variances were analysed using one-way analysis of variance (ANOVA). For data that deviated from normality and/or homoscedasticity, the Kruskal-Wallis test (non-parametric) was applied, followed by Dunn's post hoc analysis with Bonferroni correction, for pairwise comparisons. A significance level of *p < 0.05 was used for all statistical tests. Results are presented as the mean (M) ± standard deviation (SD). Bar charts were created using Prism 10 (Version 10.1.1 (270)).

## Results

### Depletion of selenium from cell culture media reduces number of progenitor cells

**Fig. 2:**
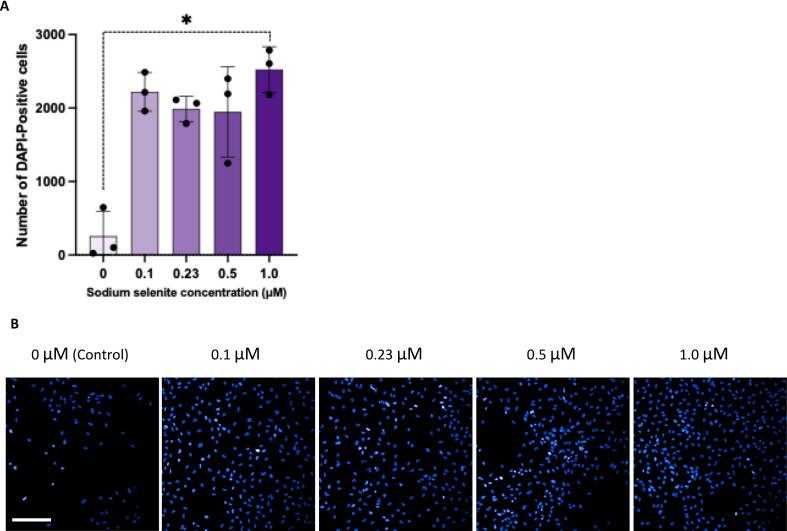
Effect of different concentrations of sodium selenite on the number of HPC0A07/03A cells in culture (DAPI-Positive cells). (A) A significant main effect of sodium selenite concentration was observed [Kruskal-Wallis test, p = 0.047]. Post hoc analyses showed a significant difference in the number of cells treated with 1.0 µM (M = 2524, SD = 311) sodium selenite compared to untreated cells (0 µM; M = 257, SD = 341) [Dunn’s test: p = 0.017]. B) Representative images (20× water objective) of cells treated with various concentrations of sodium selenite. Cells were stained with DAPI, a nuclear marker (blue). Bar charts represent the mean (M), with error bars showing the standard deviation (±SD). Adjusted p-values were calculated using the Bonferroni correction. Each data point on the bar chart represents a biological replicate (n = 3). Each biological replicate has 3 technical replicates (n = 3). Scale bar, on the bottom left represents 100 μM. *p < 0.05. (For interpretation of the references to colour in this figure legend, the reader is referred to the web version of this article.)

The impact of varying SS concentrations on cell count, assessed by DAPI-positive cells, was analysed. A main effect of SS concentration on cell count was observed (*p = 0.047*). Post hoc analysis indicated that exposure to 1.0 µM (M = 2524, SD = 311) SS led to a significant, approximately 10-fold increase in DAPI-positive cell count compared to the 0 µM (0 µM; M = 257, SD = 341) SS condition (*p = 0.017*; Fig. 2A,B). Although the other SS concentrations (0.1, 0.23, and 0.5 µM) also showed a 7- to 9-fold higher cell density compared to the 0 µM condition, these differences were not statistically significant.

### Selenium supplementation Affects integrity of hippocampal progenitor cells

**Fig. 3:**
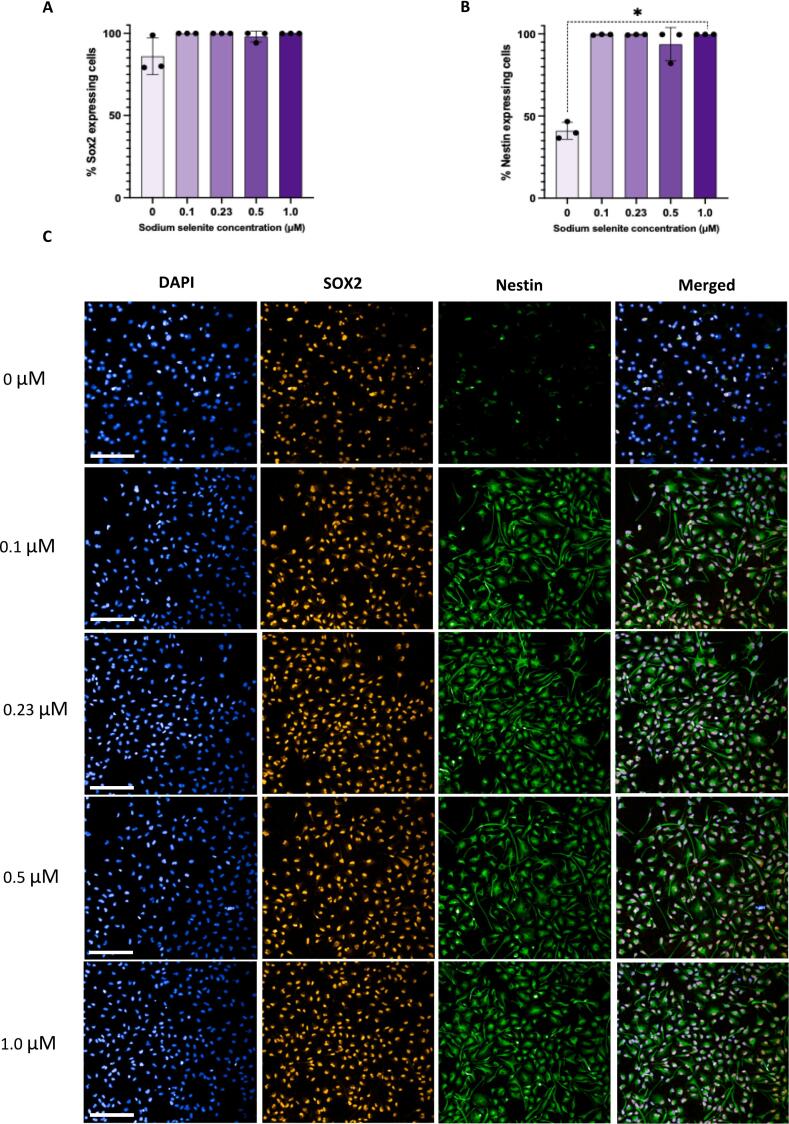
Effect of various concentrations of sodium selenite on markers of progenitor integrity in *HPC0A07/03A* cells. (A) Presence or absence of sodium selenite in the media did not significantly affect percentage of SOX2-posittive cells [Kruskal-Wallis: *p = 0.057*]. (B) A significant main effect of sodium selenite concentration on percentage of Nestin-positive cells was detected [Kruskal-Wallis test, *p = 0.021*]. Further post hoc analysis indicated that absence of sodium selenite in the media [0 µM, *M = 41.00, SD = 5.16*], significantly reduced the percentage of Nestin-positive cells, when compared to the condition with the highest concentration of sodium selenite [1 µM, *M = 99.73, SD = 0.010*, Dunn’s test, *p = 0.005*]. (C) Representative immunostaining images of cells treated with different concentrations of sodium selenite (20× water objective; Blue: DAPI, Yellow: SOX2 and Green: Nestin). Bar charts represent the Mean (M) while error bars showing the standard deviation (±SD). Adjusted p-values were calculated using the Bonferroni correction. Each data point on each the bar chart represents a biological replicate. *(n = 3).* Each biological replicate has 3 technical replicates *(n = 3).* Scale bar, on the bottom left represents 100 μM. *p < 0.05. (For interpretation of the references to colour in this figure legend, the reader is referred to the web version of this article.)

There was no significant main effect of SS concentration on the percentage of SOX2-positive cells (*p = 0.057*; Fig. 3A,C). However, a significant main effect of SS concentration on the percentage of Nestin-positive cells was observed (*p = 0.021*; Fig. 3B,C). Post hoc analysis revealed a significant difference (*p = 0.005*), between the 0 µM (M = 41.00, SD = 5.16) and 1 µM conditions (M = 99.73, SD = 0.010), with 1 µM condition showing approximately 59 % more Nestin-expressing cells compared to the control (0 µM) condition in average.

### Selenium does not affect expression of proliferation or apoptotic markers in HPCs

**Fig. 4:**
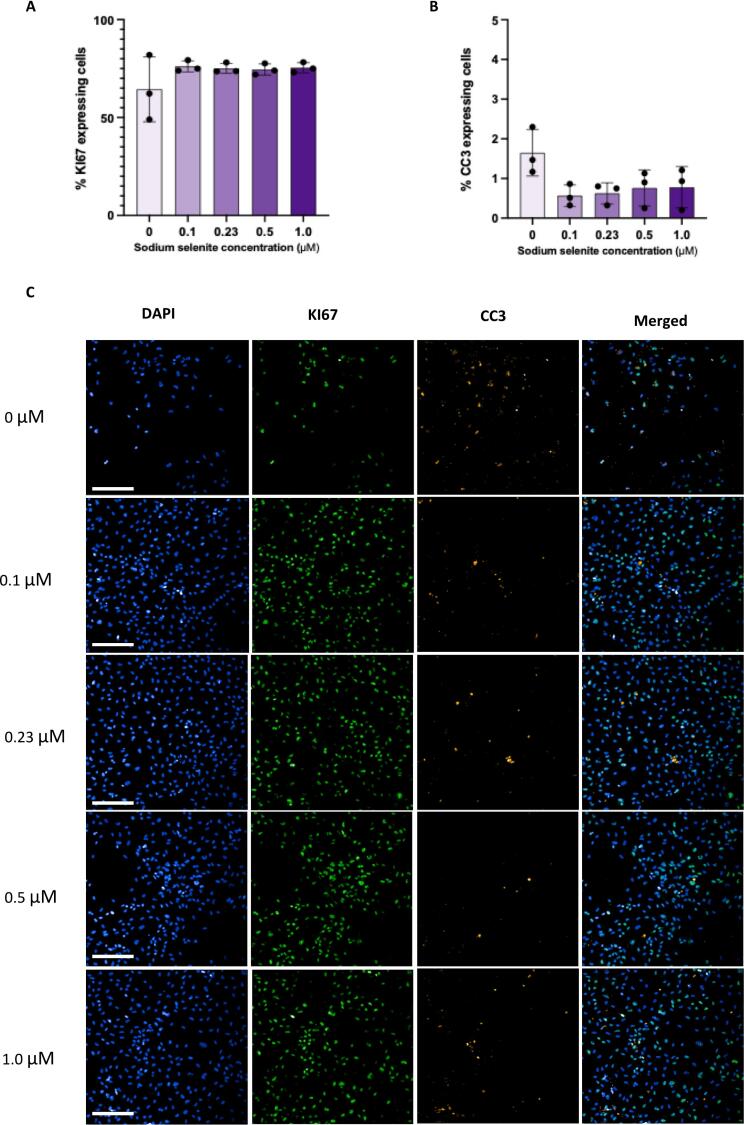
Effect of various concentrations of sodium selenite on markers of proliferation and apoptosis in *HPC0A07/03A* cells. (A) No significant difference between the conditions treated with different concentrations of sodium selenite in percentage of KI67-positve cells [Kruskal-Wallis test, *p = 0.838*] or (B) percentage of CC3-positive cells [One way ANOVA, *p = 0.069*] was observed. (C) Representative immunostaining images of cells treated with different concentrations of sodium selenite (20× water objective; Blue: DAPI, Green: KI67 and Yellow: CC3). Bar charts represent the Mean (M) while error bars showing the standard deviation (±SD). Adjusted p-values were calculated using the Bonferroni correction. Each data point on each bar chart represents a biological replicate. *(n = 3).* Each biological replicate has 3 technical replicates *(n = 3).* Scale bar, on the bottom left represents 100 μM. *p < 0.05. (For interpretation of the references to colour in this figure legend, the reader is referred to the web version of this article.)

No significant main effect of SS concentration on the percentage of KI67-expressing cells was detected (*p = 0.838;* Fig. 4.A,C). Similarly, SS concentrations did not significantly affect percentage of CC3-positive cells (*p = 0.069;* Fig. 4.B,C).

## Discussion

The effects of selenium on the proliferation and survival of mouse hippocampal progenitor cells have previously been demonstrated [27]. Until now, it remained unclear how selenium effects human hippocampal progenitor cells. In this study, we investigated the effect of selected concentrations of SS (0 μM, 0.1 μM, 0.23 μM, 0.5 μM and 1.0 μM) on proliferation, apoptosis and progenitor integrity of human HPCs. Our results indicated that absence of selenium in the culture media, significantly reduced number of cells, measured by density of DAPI-positive cells, compared to the condition with the highest concentration of selenium (Fig. 2A,B). Interestingly, in contrast to the findings from Leiter et al. [27], the observed pattern in our study, does not indicate a clear concentration-dependent increase in cell numbers. Instead, selenium treatment across a range of concentrations (0.1 – 1.0 uM), positively influenced cell numbers, with no substantial differences between the tested concentrations. While from this, it could be interpreted that selenium promotes proliferation of human HPCs, a finding in line with what Leiter et al. [27] had shown in mouse primary DG cells as well as mouse adherent hippocampal precursor cells, the results should be interpreted with caution. This is mainly due to lack of any significant differences in percentage of KI67-expressing cells between conditions (Fig. 4A,C). This finding suggests that the reduced cell number observed in the absence of selenium is not necessarily due to diminished proliferative capacity. Instead, other underlying mechanisms may be at play. One plausible explanation for the reduced cell count in absence of selenium is an increase in cell death. In fact, other studies have shown that absence of selenium from the cell culture medium, is associated with significant reduction in cell viability, in other cell types such as: T lymphocytes [33]. However, to our knowledge, this is the first report of selenium depletion-induced cell death in human HPCs.

To evaluate apoptosis mediated cell death, the expression of CC3 under different conditions was investigated. Although the SS-free condition displayed a trend towards a higher percentage of apoptotic cells compared to other conditions, this difference did not reach statistical significance (*p = 0.069*; Fig. 4B,C). This is in agreement with what was also reported in mouse adherent hippocampal precursor cells, where SS supplementation did not alter apoptosis [27]. Importantly, the lack of statistical difference in percentage of apoptotic cells between conditions, might be explained by our study's timescale. In this study, cells were fixed 48 h post-treatment, by which point a reduced cell number had already been observed in the selenium-deprived condition. This suggests that a higher percentage of apoptotic cells may have been present at earlier stages, prior to cell death, highlighting the potential for further research into the timing and dynamics of apoptotic cell death in absence of selenium.

Furthermore, it is important to note that apoptosis is not the only pathway of cell death; multiple other pathways are also associated with cell death [34,35]. For instance, selenium has also been shown to protect healthy cells from necrosis and ferroptosis [36,37]. Interestingly, findings from previous studies suggest that selenium’s role in both programmed and non-programmed cell death is context-dependent, varying with the health status of the cell. In healthy cells, selenium has been shown to inhibit apoptosis, thereby supporting cellular survival and homeostasis. Conversely, in cells with high levels of reactive oxygen species (ROS), selenium appears to induce apoptosis, acting as a potential regulatory agent by promoting cell death in the stressed cells [37]. Therefore, in order to better understand how selenium regulates survival of the human HPCs, future work should focus on assessing other modes of cell death while controlling for factors such as the cellular ROS levels.

Previous studies underscore the pivotal roles of SOX2 and Nestin in maintaining stem cell identity [38,39]. Here, to investigate how selenium influences the integrity of human HPCs, we examined the expression levels of these two markers. Our findings revealed that deprivation or exposure to varying selenium concentrations (0 µM – 1.0 µM) did not significantly alter the percentage of SOX2-positive cells (Fig. 3A,C). Conversely, the absence of selenium in the culture medium led to a pronounced reduction in the percentage of Nestin-positive cells, compared to the condition with the highest concentration of selenium (1.0 µM; Fig. 3B,C). Furthermore, to determine whether the reduction in the percentage of Nestin-positive cells is linked to the differentiation capacity of the HPCs, we examined the expression of doublecortin (DCX) and microtubule-associated protein 2 (MAP2), markers of neuroblasts and neurons, respectively, in differentiating HPCs. however, neither the absence of selenium nor its treatment with various concentrations affected the expression of either marker (Supplementary Fig. S2). This suggests that the reduction in the number of Nestin-positive cells, is not necessarily linked to the transition of HPCs from a proliferating state to differentiation in absence of selenium.

To understand why Nestin expression is affected by selenium deprivation while the percentage of SOX2-positve cells remain unchanged, it is essential to consider their distinct regulatory roles. While SOX2 is a transcription factor that is implicated in maintaining progenitor cell integrity [38,40], Nestin is an intermediate filament protein associated with cytoskeletal integrity and progenitor activity [41,42]. A potential reason for this observation could be that; selenium deprivation impairs cytoskeletal dynamics or stress-response pathways that selectively affect Nestin, without compromising the nuclear transcriptional machinery governing SOX2 expression. However, prolonged selenium deprivation might also affect SOX2 expression, as suggested by a non-significant trend observed in this study, where selenium deprivation correlated with a lower number of SOX2-positive HPCs (*p = 0.*057; Fig. 3A). This finding highlights the potential for delayed or cumulative effects of selenium deficiency on transcription factors like SOX2, warranting further investigation to clarify these dynamics.

Notably, past studies have shown that ROS accumulation can alter both the cytoskeleton and the cytoskeleton-associated proteins [43]. Given selenium’s critical role in regulating hippocampal cellular ROS levels [27], a plausible explanation for altered expression of Nestin in absence of selenium, could be that selenium deprivation could lead to elevated ROS levels, potentially disrupting cytoskeletal stability and altering Nestin expression in certain HPCs. However, to clarify whether this effect is limited to protein stability or extends to transcriptional regulation, it is essential to assess Nestin transcript levels in future studies. This approach will help determine whether selenium's absence impacts only the post-transcriptional stability of Nestin or also influences its gene expression. Additionally, future research would also greatly benefit from incorporating assays capable of directly measuring cellular ROS levels. Such tools would provide critical insights into the role of ROS in driving the observed changes in human HPCs, clarifying the connection between selenium deprivation, oxidative stress, and its downstream effects on progenitor integrity.

Given that absence of selenium resulted in reduced cell number as well as lower percentage of Nestin-expressing cells, we suggest that expression of Nestin may be essential for maintenance and proliferation of human HPCs. This is in agreement with findings in mouse NSCs [39]. Moreover, we propose that Nestin's role in the cytoskeleton may be linked to its function in self-renewal. This is supported by the observation that, in addition to the reduced percentage of Nestin-positive cells in the absence of selenium, the remaining viable Nestin-positive cells exhibited a distinct expression pattern compared to selenium-present conditions (Fig. 3C). To confirm this, further studies, such as generating a Nestin knockout model in HPCs, could provide valuable insights into the specific role of Nestin in human HPCs.

Furthermore, selenium status has been observed to progressively decline with advancing age [44]. Meanwhile, maintained or increased levels of selenium have been shown to have anti-aging properties [26]. Past research has linked insufficient selenium levels to higher risk of developing neurodegenerative diseases such as Alzheimer’s disease [45]. Importantly, in vivo studies have demonstrated that selenium supplementation can reverse learning and memory impairments in mouse models of physiological aging [27], highlighting the importance of selenium in healthy aging and cognition. Given the findings of our study, we propose that selenium supplementation can potentially aid, maintain human HPC integrity and boost their survival, features which are negatively impacted as a result of aging [6,13]. While we believe our study offers valuable insights regarding the importance of selenium in maintaining homeostasis in human HPCs, it should be noted that our findings are derived from an in vitro model. Therefore, any generalisations to complex in vivo systems should be done with caution. Additionally, considering that the HPCs used in this study are acquired from female fetal tissue, the applicability of our results to both males and females requires careful consideration. This is mainly due sex-dependent differences in the neurogenic process [46] as well as selenium metabolism and uptake [47].

Lastly, while selenium supplementation as a dietary factor appears to be an efficient way to promote healthy aging, further research is required to uncover whether PE can also elevate selenium levels in humans and how this potential increase might influence neurogenesis and, consequently, cognition.

## Conclusion

Overall, we demonstrated that treatment with selenium, a trace element with antioxidant properties, positively impacts survival and integrity of human hippocampal progenitor cells. However, further studies are needed to (i) uncover the underlying mechanisms driving these changes and (ii) identify the connection between selenium supplementation and changes in neurogenesis-dependent cognition in humans.

## CRediT authorship contribution statement

**Sahand Farmand:** Writing – review & editing, Writing – original draft, Methodology, Investigation, Formal analysis, Data curation, Conceptualization. **Emaan Ahmed:** Methodology, Investigation. **Hadisa Azizi Zawar:** Methodology, Investigation. **Sandrine Thuret:** Writing – review & editing, Supervision, Resources, Funding acquisition, Conceptualization.

## Funding

This work was supported by the Reta Lila Weston Trust and the UKRI Ageing Across the Lifecourse Networks Biotechnology and Biological Sciences Research Council (BBSRC) (BB/W018381/1).

## Declaration of competing interest

The authors declare that they have no known competing financial interests or personal relationships that could have appeared to influence the work reported in this paper.

## Data Availability

The full raw research datasets from this manuscript will be deposited and linked to this manuscript via the open access repository Figshare LLP upon acceptance.
