# Supplementary material for: Selenium deficiency negatively affects survival and integrity of human hippocampal progenitor cells
Source: Aging Brain. 2025 Apr 30;7:100138. doi: 10.1016/j.nbas.2025.100138 (PMC12084072; doi:10.1016/j.nbas.2025.100138)
Supplement: Supplementary Data 1 [file mmc1.docx]

**Supplementary**

**Differentiation assay timeline**

**
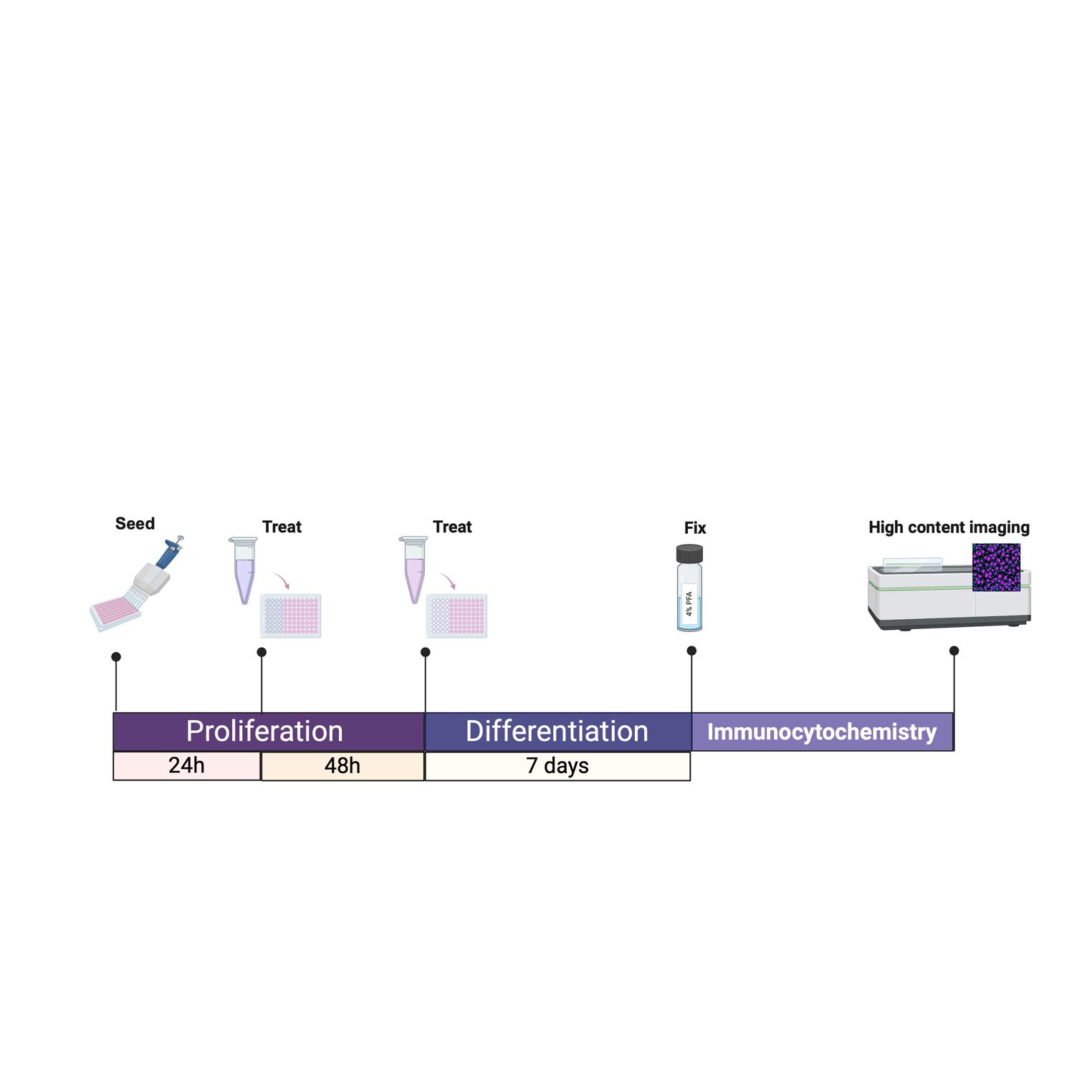
**

**Figure S1.** Schematic of the differentiation assays for in vitro supplementation of HPC0A07/03A cells with sodium selenite. To assess markers associated with differentiation using immunocytochemistry, cells were seeded in sodium selenite-free media and allowed to proliferate for 24h. The sodium selenite-free media was then removed from the wells, and cells were treated with various concentrations of sodium selenite (0 μM, 0.1 μM, 0.23 μM, 0.5 μM and 1.0 μM). After a further 48h of proliferation, cells were washed twice with differentiation medium (30 minutes each time) and were treated again, with selenium treatments made in differentiation media. Cells were then allowed to differentiate for 7 days and were then fixed using 4% PFA, immunocytochemistry was performed, followed by high content imaging using the Opera Phenix. (Created with BioRender).

**Selenium treatment and differentiation markers**

**A**

**B**

**C**


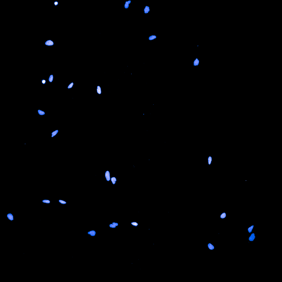

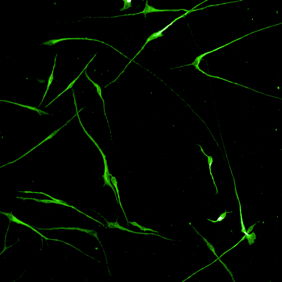

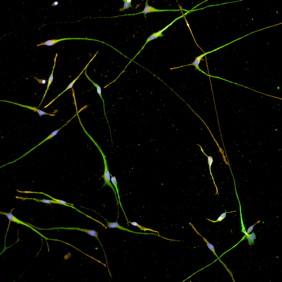

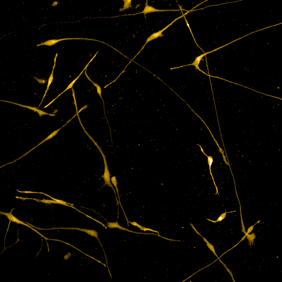

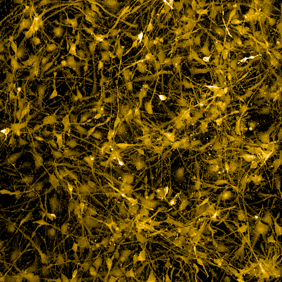

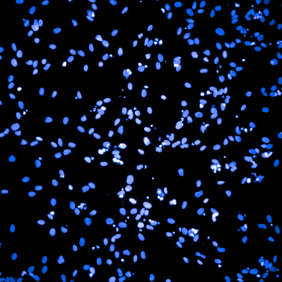

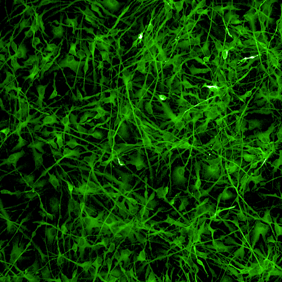

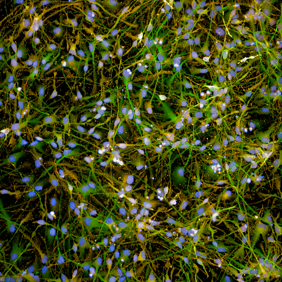

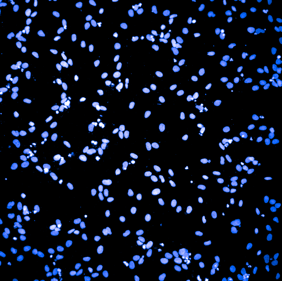

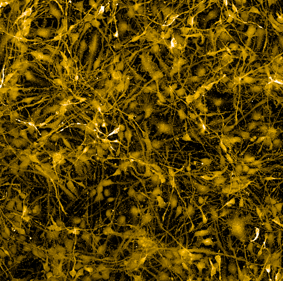

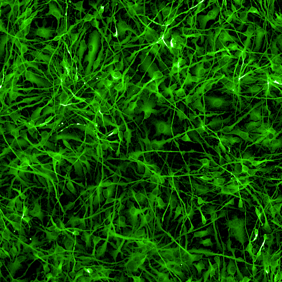

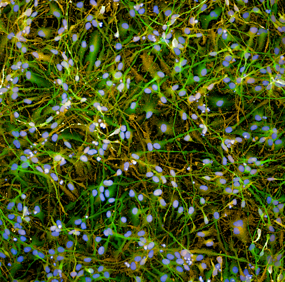

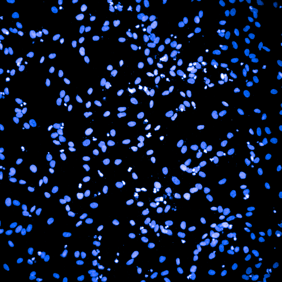

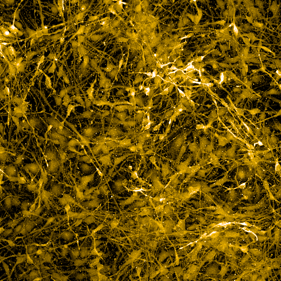

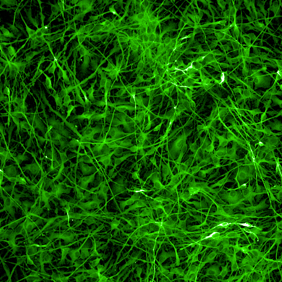

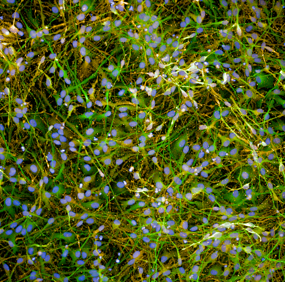

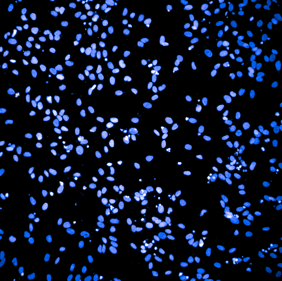

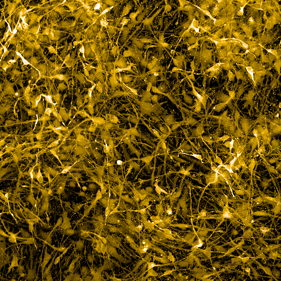

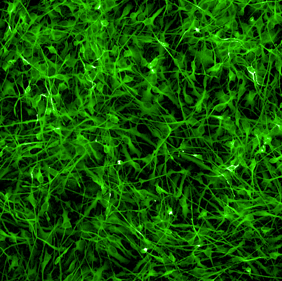

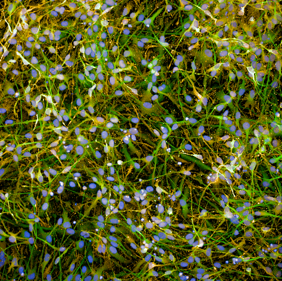


**DAPI**

**DCX**

**MAP2**

**Merged**

0.1 µM

0 µM

0.23 µM

0.5 µM

1.0 µM

**Figure S2.** Effect of various concentrations of sodium selenite on markers of neuroblasts (DCX) and neuronal differentiation (MAP2) in HPC0A07/03A cells. **A)** No significant difference between the conditions treated with different concentrations of sodium selenite in percentage of DCX-positive cells [One way ANOVA, p = 0.782] or **B)** percentage of MAP2-positive cells [One way ANOVA, p = 0.236] was observed. **C)** Representative immunostaining images of cells treated with different concentrations of sodium selenite (20X water objective; Blue: DAPI, Yellow: DCX, Green: MAP2). Bar charts represent the Mean (M) while error bars showing the standard deviation (±SD). Adjusted p-values were calculated using the Bonferroni correction. Each data point on each bar chart represents a biological replicate. (n = 3). Each biological replicate has 3 technical replicates (n = 3). Scale bar, on the bottom left represents 100 μM. *p<0.05.
